# Air pollution, residential greenness, and metabolic dysfunction biomarkers: analyses in the Chinese Longitudinal Healthy Longevity Survey

**DOI:** 10.1186/s12889-022-13126-8

**Published:** 2022-05-04

**Authors:** Linxin Liu, Lijing L. Yan, Yuebin Lv, Yi Zhang, Tiantian Li, Cunrui Huang, Haidong Kan, Junfeng Zhang, Yi Zeng, Xiaoming Shi, John S. Ji

**Affiliations:** 1 Vanke School of Public Health, Tsinghua University, Beijing, China; 2 Global Heath Research Center, Duke Kunshan University, Kunshan, China; 3 School of Public Health, Wuhan University, Wuhan, China; 4 Institute for Global Health and Development, Peking University, Beijing, China; 5 China CDC Key Laboratory of Environment and Population Health, National Institute of Environmental Health, Chinese Center for Disease Control and Prevention, Beijing, China; 6 School of Public Health, Fudan University, Shanghai, China; 7 Nicholas School of the Environment and Duke Global Health Institute, Duke University, Durham, NC USA; 8 Center for Healthy Aging and Development Studies, National School of Development, Peking University, Beijing, China; 9 Center for the Study of Aging and Human Development, Duke Medical School, Durham, NC USA; 10 Center for Global Health, School of Public Health, Nanjing Medical University, Nanjing, China

**Keywords:** Air pollution, Greenness, Interaction, Metabolic syndrome, Aging

## Abstract

**Background:**

We hypothesize higher air pollution and fewer greenness exposures jointly contribute to metabolic syndrome (MetS), as mechanisms on cardiometabolic mortality.

**Methods:**

We studied the samples in the Chinese Longitudinal Healthy Longevity Survey. We included 1755 participants in 2012, among which 1073 were followed up in 2014 and 561 in 2017. We used cross-sectional analysis for baseline data and the generalized estimating equations (GEE) model in a longitudinal analysis. We examined the independent and interactive effects of fine particulate matter (PM_2.5_) and Normalized Difference Vegetation Index (NDVI) on MetS. Adjustment covariates included biomarker measurement year, baseline age, sex, ethnicity, education, marriage, residence, exercise, smoking, alcohol drinking, and GDP per capita.

**Results:**

At baseline, the average age of participants was 85.6 (SD: 12.2; range: 65–112). Greenness was slightly higher in rural areas than urban areas (NDVI mean: 0.496 vs. 0.444; range: 0.151–0.698 vs. 0.133–0.644). Ambient air pollution was similar between rural and urban areas (PM_2.5_ mean: 49.0 vs. 49.1; range: 16.2–65.3 vs. 18.3–64.2). Both the cross-sectional and longitudinal analysis showed positive associations of PM_2.5_ with prevalent abdominal obesity (AO) and MetS, and a negative association of NDVI with prevalent AO. In the longitudinal data, the odds ratio (OR, 95% confidence interval-CI) of PM_2.5_ (per 10 μg/m^3^ increase) were 1.19 (1.12, 1.27), 1.16 (1.08, 1.24), and 1.14 (1.07, 1.21) for AO, MetS and reduced high-density lipoprotein cholesterol (HDL-C), respectively. NDVI (per 0.1 unit increase) was associated with lower AO prevalence [OR (95% CI): 0.79 (0.71, 0.88)], but not significantly associated with MetS [OR (95% CI): 0.93 (0.84, 1.04)]. PM_2.5_ and NDVI had a statistically significant interaction on AO prevalence (*p*_interaction_: 0.025). The association between PM_2.5_ and MetS, AO, elevated fasting glucose and reduced HDL-C were only significant in rural areas, not in urban areas. The association between NDVI and AO was only significant in areas with low PM_2.5_, not under high PM_2.5_.

**Conclusions:**

We found air pollution and greenness had independent and interactive effect on MetS components, which may ultimately manifest in pre-mature mortality. These study findings call for green space planning in urban areas and air pollution mitigation in rural areas.

**Supplementary Information:**

The online version contains supplementary material available at 10.1186/s12889-022-13126-8.

## Background

Metabolic syndrome (MetS) is a risk factor for morbidity and mortality. Specifically, it is a group of pathologic conditions that precede non-communicable diseases, including cardiovascular disease (CVD) and diabetes [1]. It has become a global problem with the increasing prevalence in both developed and developing countries [2]. There are plenty of amenable causes of MetS. An increasing number of studies have been focusing on environmental determinants.

Fine particulate matter (PM_2.5_) is an independent risk factor for mortality in many locations and exposure levels [3]. PM_2.5_ has been implicated in causing systemic inflammation and altered metabolism of lipids and glucose [4–6]. At the same time, living in areas with higher greenness is associated with a reduced risk of mortality and ﻿cardiovascular disease [7]. However, there was no established evidence on the association between PM_2.5_ and MetS according to current controversial findings in various countries [8, 9]. A limited number of research findings in China were inconsistent [10, 11]. Compared to air pollution, much less attention has been paid to greenness and MetS worldwide, especially for the older adults aged 80 or older, and there was also little agreement [12–14]. Some prior findings showed combined or synergistic effects of PM_2.5_ and greenness on mortality [15, 16]. No studies looked at their interaction on MetS based on our knowledge.

The relationship between air pollution and residential greenness can be complex and need additional analyses for generalizability in different climates, income levels, and places with varying population density. A recent study based on a Canadian cohort of 2.4 million individuals found adjustment of greenness attenuated the effect of PM_2.5_. The effect of air pollution on cardiovascular mortality was the largest in places with the least greenness. Studies that do not account for greenness may overstate the harmful effect of air pollution on mortality [15]. In a seven metropolitan cities study in South Korea, the effect of PM_10_ was higher in areas of lower greenness for cardiovascular-related mortality, but not for non-accidental mortality and respiratory-related mortality [17]. A cohort study spanning 22 provinces in China of elderly individuals found that people living in urban areas experienced higher health benefits of greenness. People living in rural regions were more likely to be harmed by air pollution [16]. Not all studies found a significant interaction between greenness and air pollution. An Israel-based study found the incorporation of greenness into the PM_2.5_ model did not improve the cardiovascular disease predictions for stroke and myocardial infarction, although air pollution and greenness had strong independent effects on these outcomes [18]. As for MetS, KORA F4/FF4 cohort in Germany and Whitehall II study in the UK found the association between greenness and MetS was reversed and became positive after adjusting for PM_2.5_ in the model. In contrast, 33 Communities Chinese Health Study (33CCHS) in China found this association was only partly attenuated after adjusting for air pollution [12–14].

Large uncertainty still exists about the pattern and mechanisms of greenness and air pollution impact on MetS. With the rapid urbanization and population aging in developing countries, including China, the role of these environmental determinants is yet to be determined. Using a cohort of older adults in eight regions in China, we aim to (1) estimate the prevalence of MetS and its components based on measured biomarkers, (2) determine the independent effects of PM_2.5_ and greenness on metabolic syndrome biomarkers, (3) assess the interactive effect of PM_2.5_ and greenness, and (4) to assess effect modification by age, gender, and urban versus rural regions. These analyses are anticipated to generate insights that can improve our limited understanding of whether and how the two important environmental factors related to urbanization affect metabolic syndrome, a health problem with increasing prevalence in rapidly developing parts of the world.

## Methods

### Study population

We used data from the sub-cohort of the Chinese Longitudinal Healthy Longevity Survey: Healthy Ageing and Biomarkers Cohort Study (HABCS). The study collected blood samples for biomarker examinations during 2008 to 2017 in eight places designated as longevity areas (Laizhou City of Shandong Province, Xiayi County of Henan Province, Zhongxiang City of Hubei Province, Mayang County of Hunan Province, Yongfu County of Guangxi Autonomous Area, Sanshui District of Guangdong Province, Chengmai County of Hainan Province and Rudong County of Jiangsu Province). The published cohort profile described the study design and sample method [19]. The waist circumference was measured since 2012. We set the study baseline at 2012 and excluded 85 participants aged younger than 65, 286 participants with missing biomarker value, 91 participants with missing NDVI or PM_2.5_ value, and 222 participants with missing covariates value (Fig. S1). We finally included 1755 participants at baseline. During 2012–2017, 1115 participants were followed up at least twice, and 519 participants were followed up three times.

### Air pollution and residential greenness measurements

Ground-level PM_2.5_ concentrations were estimated by the Atmospheric Composition Analysis Group. They combined aerosol optical depth retrievals from the National Aeronautics and Space Administration’s Moderate Resolution Imaging Spectroradiometer, Multi-angle Imaging SpectroRadiometer, and Sea-viewing Wide field-of-view Sensor satellite instruments; vertical profiles derived from the GEOS-Chem chemical transport model; and calibration to ground-based observations of PM_2.5_ using geographically weighted regression [20]. The resultant PM_2.5_ concentration estimates were highly consistent (R^2^ = 0.81) with out-of-sample cross-validated PM_2.5_ concentrations from monitors. We matched the annual average PM_2.5_ concentrations in a 1 km × 1 km grid to each participant’s residence [21].

We calculated Normalized Difference Vegetation Index (NDVI) with a 500-m radius around each participant’s residence to quantify greenness exposure. We used satellite images from the Moderate-Resolution Imaging Spectro-Radiometer (MODIS) in the National Aeronautics and Space Administration’s Terra Satellite. The NDVI calculation formula is near-infrared radiation minus visible radiation divided by near-infrared radiation plus visible radiation, ranging from − 1.0 to 1.0, with larger values indicating higher vegetative density levels. There are two NDVI values for January, April, July, and October between 2008 and 2014 in our database to reflect the seasonal variation of greenness. We linked NDVI imagery to the longitude and latitude of each residential address and calculated greenness in 500 m radii.

We matched time-varying annual PM_2.5_ and NDVI of 2008–2014 to the data. We calculated the average value of one-year, three-year, and five-year exposure time windows as long-term cumulative exposures measurements. We used the same exposure results as the 2014 wave for the 2017 wave since we lacked the environmental exposure data from 2014 to 2017.

### Biomarker measurements

The participants provided the blood sample at the same time as the interview time in 2012, 2014, and 2017. The medical technician tested blood plasma biomarkers included fasting glucose, glycated serum protein (GSP), total cholesterol (TC), triglyceride (TG), and high-density lipoprotein cholesterol (HDL-C) using an Automatic Biochemistry Analyzer (Hitachi 7180, Japan) with commercially available diagnostic kits (Roche Diagnostic, Mannheim, Germany) at Capital Medical University in Beijing. Low-density lipoprotein cholesterol (LDL-C) was calculated using the formula of Friedewald et al.: LDL-C = TC-(HDL-C)-TG/5 [22].

Trained medical staff performed anthropometric measurements for the participants, including waist circumference, and two blood pressure measurements with at least a one-minute interval between them. We used the mean value of the two blood pressure measurements.

### Definition of metabolic syndrome (MetS) and components

We defined the MetS using the Adult Treatment Panel III of the National Cholesterol Education Program (ATP III) guidelines, modified in accordance with the waist circumference cutoff points proposed by World Health Organization (WHO) for Asian populations (modified ATP III). It was defined as the presence of at least three of the following criteria: elevated fasting glucose (fasting glucose≥100 mg/dL), abdominal obesity (AO: Waist circumference ≥ 90 cm for males and ≥ 80 cm for females), hypertension (SBP ≥ 130/DBP ≥ 85 mmHg), hypertriglyceridemia (TG ≥ 150 mg/dL), and reduced HDL-C (HDLC< 40 mg/dL for males and < 50 mg/dL for females) [23, 24]. We also did sensitivity analysis for the MetS defined by the Joint Interim Societies [25].

### Baseline covariates

We categorized the ethnicity as Han Chinese or ethnic minorities. We used years in schools as a measure of literacy level. We classified marital status into two categories: currently married and living with the spouse, or not married (widowed/separated/divorced/never married/married but not living with the spouse). We classified city and town as “Urban”, and village as “Rural.” We firstly divided the regular exercise, smoking, and alcohol drinking status into three categories: “Current,” “Former,” and “Never”. For example, participants were asked, “do you do exercise regularly at present (planned exercise like walking, playing balls, running and so on)?” and/or “did you do exercise regularly in the past?”. We defined the regular exercise status as “Current” for participants who answered “Yes” to the first question, “Former” for who answered “No” to the first question and “Yes” to the second question, and “Never” for who answered “No” to both two questions. Then we further quantified the current smoker based on the number of times smoke (or smoked) per day: < 20 times/day and ≥ 20 times/day. We also quantified the current alcohol drinker based on the kind of alcohol and how much they drank per day. The unit of alcohol was a Chinese unit of weight called ‘Liang’ [50 g (g)]. The level of alcohol consumption was calculated as drinks of alcohol per day, based on the beverage type and amount, assuming the following alcohol content by volume (v/v) typically seen in China: strong liquor 53%, weak liquor 38%, grape wine 12%, rice wine 15%, and beer 4% [26]. A standard drink was equal to 14.0 g of pure alcohol according to the criterion of the Center for Disease Control and Prevention in the USA, and moderate drinking is up to 1 drink per day for women and up to 2 drinks per day for men according to Dietary Guidelines for Americans 2015–2020. Therefore, we defined those who drank equal or less than 14 g pure alcohol per day for the female or 28 g per day for the male as light drinkers, otherwise heavy drinkers. We collected Gross Domestic Product (GDP) per capita by county/district from the local statistical yearbook.

### Statistical analysis

We described univariate statistics of our exposure, outcome variables, and covariates in eight areas. We built the multivariate logistic regression model in the cross-sectional analysis to analyze the association between residential environment (residential greenness and ambient air pollution) and baseline MetS and each component. For the longitudinal analysis, we used generalized estimating equations (GEE) to assess the association between the repeatedly measured residential environment and the repeatedly measured metabolic biomarkers. For each biomarker: firstly, we built the single exposure model to regress only one environment factor on the biomarker; Second, we built the two-exposure model to regress both greenness and air pollution on the biomarker; Third, we added the product term of centered greenness and air pollution (NDVI×PM_2.5_) in the model to assess their interaction and one exposure’s association with the outcome under another exposure’s mean level. We adjusted for biomarker measurement year, baseline age, sex, ethnicity, education, marriage, residence, exercise, smoking, alcohol drinking, and GDP per capita in these models. Considering gender difference plays a vital role in the health of the old population, we further examined the greenness, air pollution, and gender three-way interaction by adding the term “NDVI×PM_2.5_ × Sex” in the model. We performed sensitivity analyses using environment exposure of different time windows (1 year or five-year average NDVI or PM_2.5_). Given the selection bias due to lost to follow-up, we also built models for those with at least one follow-up. We conducted stratified analyses based on age, sex, and residence to test the possible modification. We set the nominal significance level at 0.05. We used R 4.0.0 to run all the analyses.

## Results

### Population characteristics and environmental exposure level

We studied 1755 participants aged 65 to 112 years old, with a mean age of 85 (SD:12.2); 53.8% were female. Most were Han participants (92.3%), lived in rural areas (83.1%), never had regular exercise (81.9%), never smoked (75.4%), and never drank alcohol (77.9%). There were 370 (21.1%) participants who fit the criteria for MetS, 583 (33.2%) for abdominal obesity (AO), 307 (17.5%) for elevated fasting glucose, 1285 (73.2%) for hypertension, 157 (8.9%) for hypertriglyceridemia, and 679 (38.7%) for reduced HDL-C (Table 1). Those who were lost of follow-up were older, more likely to be female, living in areas with higher GDP, not currently married, and without formal education (Table S1).

**Table 1:** Baseline population characteristics

Variables	Residence		Overall
	Urban (***N*** = 296)	Rural (***N*** = 1459)	(***N*** = 1755)
**3-year average NDVI: mean (SD) (0.1 unit)**	4.44 (1.25)	4.96 (0.83)	4.88 (0.94)
**3-year average PM**_**2.5**_**: mean (SD) (10 μg/m**^**3**^**)**	4.91 (1.14)	4.90 (1.60)	4.90 (1.53)
**GDP per capita in 2012: mean (SD) (10,000 RMB)**	4.77 (4.85)	4.27 (3.64)	4.35 (3.87)
**Sex:** ***n*(%)** Male	127 (42.9)	683 (46.8)	810 (46.2)
**Age: mean (SD)**	84.6 (11.9)	85.8 (12.3)	85.6 (12.2)
**Schooling year:** ***n*(%)**			
No formal education	168 (56.8)	918 (62.9)	1086 (61.9)
1–6 years education	88 (29.7)	417 (28.6)	505 (28.8)
> 6 years education	40 (13.5)	124 (8.5)	164 (9.3)
**Ethnicity:** ***n*(%)** Han	269 (90.9)	1351 (92.6)	1620 (92.3)
**Marriage:** ***n*(%)** Currently married	115 (38.9)	563 (38.6)	678 (38.6)
**Exercise:** ***n*(%)**			
Never	238 (80.4)	1199 (82.2)	1437 (81.9)
Former	4 (1.4)	37 (2.5)	41 (2.3)
Current	54 (18.2)	223 (15.3)	277 (15.8)
**Smoking:** ***n*(%)**			
Never	244 (82.4)	1079 (74.0)	1323 (75.4)
Former	17 (5.7)	128 (8.8)	145 (8.3)
< 20 times/day	21 (7.1)	141 (9.7)	162 (9.2)
≥ 20 times/day	14 (4.7)	111 (7.6)	125 (7.1)
**Alcohol:** ***n*(%)**			
Never	245 (82.8)	1123 (77.0)	1368 (77.9)
Former	20 (6.8)	80 (5.5)	100 (5.7)
≤ 14 g/d(female) 28(male)	9 (3.0)	91 (6.2)	100 (5.7)
> 14 g/d(female) 28(male)	22 (7.4)	165 (11.3)	187 (10.7)
**TC: mean (SD) (mmol/L)**	4.30 (0.954)	4.28 (0.981)	4.29 (0.976)
**LDL-C: mean (SD) (mmol/L)**	2.43 (0.821)	2.57 (0.821)	2.54 (0.822)
**TG: median (P25-P75) (mg/dL)**	87 (61–118)	70 (51–98)	73 (52–102)
**HDL-C: mean (SD) (mg/dL)**	51.3 (15.2)	49.8 (13.7)	50.1 (14.0)
**Waist circumference: mean (SD) (centimeter)**	79.6 (11.4)	79.7 (10.8)	79.6 (10.9)
**Fasting glucose: median (P25-P75) (mg/dL)**	76 (54–91)	80 (68–93)	80 (67–92)
**SBP: mean (SD) (mmHg)**	141 (21.1)	140 (23.1)	141 (22.8)
**DBP: mean (SD) (mmHg)**	82.8 (11.2)	80.8 (12.1)	81.1 (11.9)
**Abdominal obesity:** ***n*(%)** Yes	107 (36.1)	476 (32.6)	583 (33.2)
**Elevated fasting glucose:** ***n*(%)** Yes	41 (13.9)	266 (18.2)	307 (17.5)
**Hypertension:** ***n*(%)** Yes	225 (76.0)	1060 (72.7)	1285 (73.2)
**Hypertriglyceridemia:** ***n*(%)** Yes	40 (13.5)	117 (8.0)	157 (8.9)
**Reduced HDL-C:** ***n*(%)** Yes	112 (37.8)	567 (38.9)	679 (38.7)
**Mets:** ***n*** **(%)** Yes	67 (22.6)	303 (20.8)	370 (21.1)

**Fig. 1:**
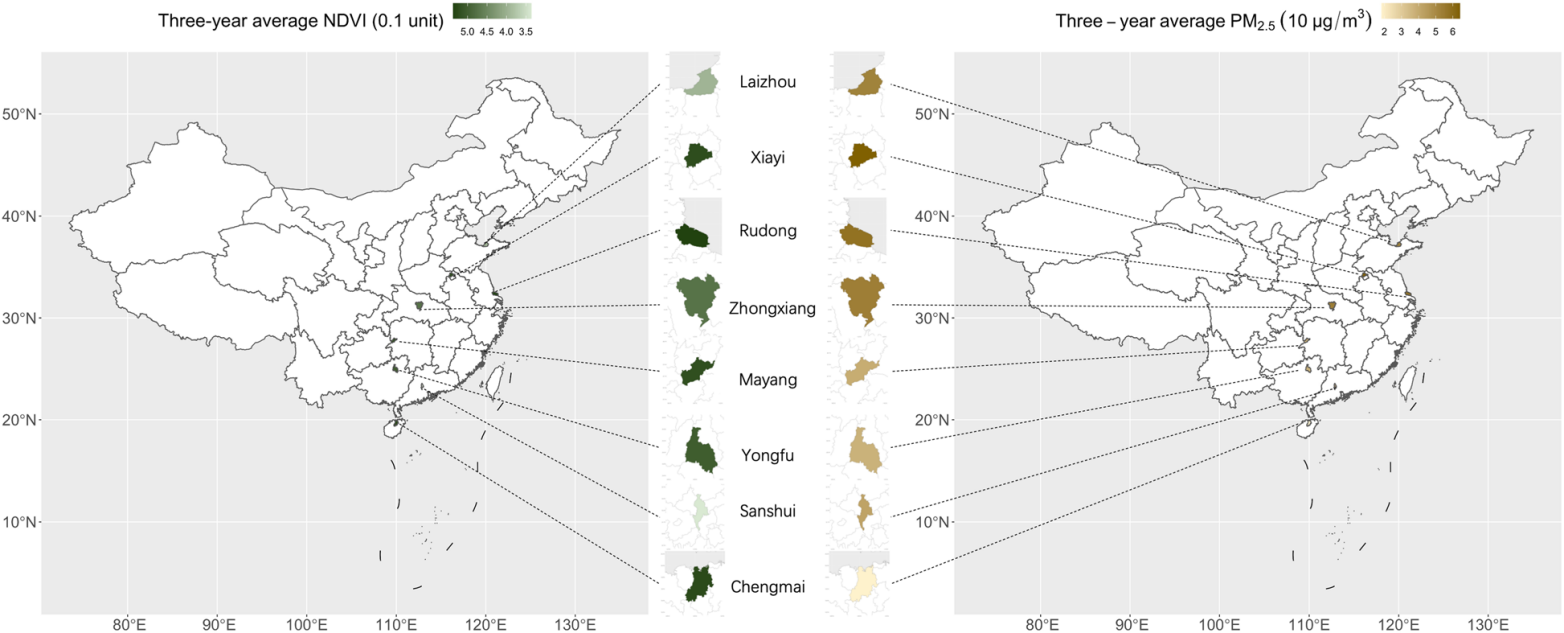
The NDVI and PM2.5 level in the eight sample districts. Note: We used “ggplot2” and “sf” packages in R 4.0.0 (URL https://www.R-project.org/) to draw the map

PM_2.5_ was not associated with NDVI (Pearson correlation coefficient: 0.0004; *p* > 0.05). The three-year NDVI (0.1 unit) of the rural area was slightly higher than the urban area (mean: 4.96 vs. 4.44; range: 1.51–6.98 vs. 1.33–6.44), and the mean of three-year PM_2.5_ (10 μg/m^3^) were almost the same in the rural and urban areas (mean: 4.90 vs. 4.91; range: 1.62–6.53 vs. 1.83–6.42) of our sample (Table 1). The mean of the three-year NDVI (0.1 unit) of the eight counties was 4.88 (SD: 0.94), ranging from 3.36 (0.81) in Sanshui to 5.37 (0.59) in Rudong. The mean of three-year PM_2.5_ (10 μg/m^3^) of the eight areas was 4.90 (SD: 1.53), ranging from 1.83 μg/m^3^ (SD: 0.03) in Chengmai to 6.42 μg/m^3^ (SD: 0.02) in Xiayi (Fig. 1, Table S2).

### Environmental exposure and MetS

In both the cross-sectional and longitudinal analyses, higher PM_2.5_ was associated with higher odds of MetS [OR (95%CI): 1.17 (1.07, 1.28) and 1.16 (1.08, 1.24) respectively], and the association between NDVI and MetS tended to be negative but was not statistically significant [OR (95%CI): 0.94 (0.81, 1.09) and 0.93 (0.84, 1.04) respectively]. These associations did not change when adding both PM_2.5_ and NDVI in the model, and there was no significant interaction between PM_2.5_ and NDVI on MetS (Table 2 & Table S3).

**Table 2:** The association between the greenness and air pollution with the metabolic syndrome and the components (Binary outcome) in the longitudinal analysis^a^

Outcome	Exposure	Greenness single exposure model (0.1 unit increase of NDVI)		PM_2.5_ single exposure model (10 μg/m^3^ increase of PM_2.5_)		Greenness & PM_2.5_ two exposure model		Centered Greenness & PM_2.5_ interaction model		
		OR (95% CI)	*p* value	OR (95% CI)	*p* value	OR (95% CI)	*p* value	Beta	std error	*p* value
Abdominal obesity	NDVI	0.79 (0.71, 0.88)	< 0.001			0.81 (0.73, 0.90)	< 0.001	−0.210	0.056	< 0.001
Abdominal obesity	PM_2.5_			1.19 (1.12, 1.27)	< 0.001	1.18 (1.11, 1.26)	< 0.001	0.199	0.037	< 0.001
Abdominal obesity	NDVIPM_2.5_							−0.088	0.039	0.025
Elevated fasting glucose	NDVI	0.93 (0.84, 1.04)	0.192			0.94 (0.85, 1.05)	0.277	−0.054	0.055	0.332
Elevated fasting glucose	PM_2.5_			1.06 (0.99, 1.13)	0.071	1.06 (0.99, 1.13)	0.096	0.027	0.037	0.464
Elevated fasting glucose	NDVI^*^PM_2.5_							0.076	0.042	0.073
Hypertension	NDVI	0.99 (0.89, 1.11)	0.902			0.99 (0.89, 1.10)	0.872	−0.008	0.055	0.885
Hypertension	PM_2.5_			0.99 (0.93, 1.06)	0.762	0.99 (0.93, 1.06)	0.75	−0.015	0.039	0.696
Hypertension	NDVI^*^PM_2.5_							0.012	0.049	0.808
Hypertriglyceridemia	NDVI	1.01 (0.89, 1.16)	0.843			1.02 (0.89, 1.17)	0.752	0.042	0.074	0.574
Hypertriglyceridemia	PM_2.5_			1.04 (0.95, 1.13)	0.449	1.04 (0.95, 1.14)	0.43	−0.026	0.049	0.592
Hypertriglyceridemia	NDVI^*^PM_2.5_							0.158	0.056	0.005
Reduced HDL-C	NDVI	0.98 (0.88, 1.08)	0.646			1.00 (0.90, 1.11)	0.998	0.001	0.055	0.981
Reduced HDL-C	PM_2.5_			1.14 (1.07, 1.21)	< 0.001	1.14 (1.07, 1.21)	< 0.001	0.095	0.036	0.009
Reduced HDL-C	NDVI^*^PM_2.5_							0.095	0.041	0.019
MetS	NDVI	0.93 (0.84, 1.04)	0.213			0.96 (0.86, 1.07)	0.462	−0.042	0.057	0.461
MetS	PM_2.5_			1.16 (1.08, 1.24)	< 0.001	1.15 (1.07, 1.24)	< 0.001	0.121	0.040	0.003
MetS	NDVI^*^PM_2.5_							0.053	0.043	0.213

^a^All models adjusted for biomarker measurement year, baseline age, sex, ethnicity, education, marriage, residence, exercise, smoking, alcohol drinking, and GDP per capita

### Environmental exposure and MetS components

In both the cross-sectional and longitudinal analyses, higher PM_2.5_ was associated with higher odds of AO [OR (95%CI): 1.25 (1.16, 1.36) and 1.19 (1.12, 1.27) respectively], while higher NDVI was associated with lower odds of AO [OR (95% CI): 0.81 (0.71, 0.92) and 0.79 (0.71, 0.88) respectively] (Table 2 & Table S3). In addition, higher PM_2.5_ was associated with higher waist circumference [mean difference (95% CI): 1.12 (0.83, 1.40)] while higher NDVI was associated with lower waist circumference [mean difference (95% CI): − 1.21 (− 1.76, − 0.66)] (Table S4).

For the lipids, higher PM_2.5_ was only associated with higher odds of reduced HDL-C [OR (95%CI): 1.14 (1.07, 1.21)] in the longitudinal analyses. There were no significant association between PM_2.5_ and TG or hypertriglyceridemia, or between NDVI and TG, HDL-C, hypertriglyceridemia or reduced HDL-C. Besides, PM_2.5_ and NDVI were both negatively associated with TC and LDL-C (Table 2, Table S4). The association between PM_2.5_ and elevated fasting glucose were not statistically significant in either cross-sectional or longitudinal analyses [OR (95%CI): 1.08 (0.99, 1.19) and 1.06 (0.99, 1.13) respectively]. NDVI showed a negative association with the odds of elevated fasting glucose only in the cross-sectional analyses [OR (95%CI): 0.84 (0.72, 0.99)] (Table 2, Table S3). Both PM_2.5_ and NDVI were not associated with hypertension in either cross-sectional or longitudinal analyses. These results also persisted in the two-exposure model (Table 2, Table S3 and S4).

### Sensitivity analyses

Using the one-year and five-year average exposure window, the above associations persisted except for that the positive association between one-year PM_2.5_ and odds of elevated fasting glucose became statistically significant (Table S5). Among those with at least one follow-up, the results did not change significantly either (Table S6). The findings based on the Joint Interim Societies definition of MetS were also similar (Table S7).

### Possible effect modification

**Fig. 2:**
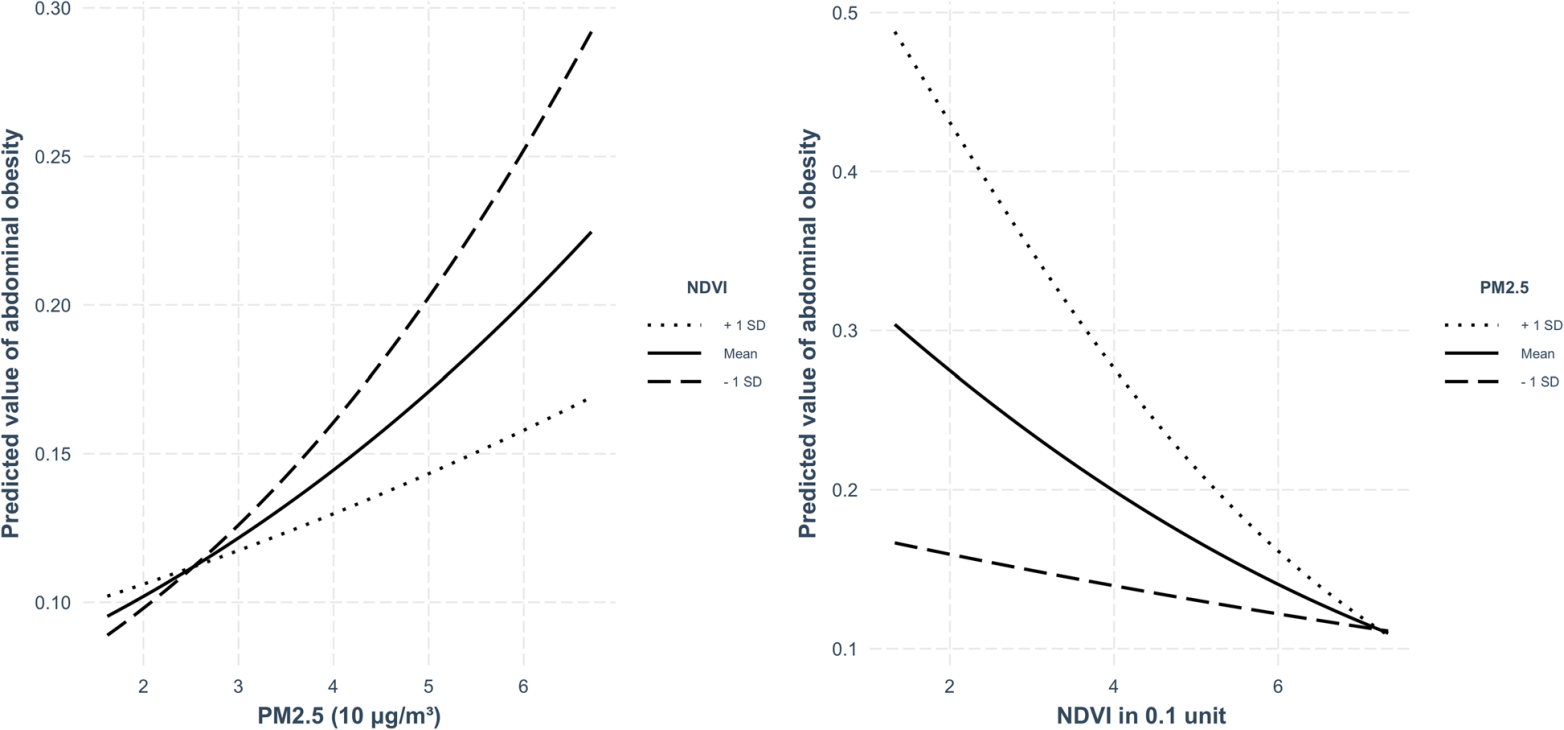
The interaction model of PM_2.5_ and NDVI on abdominal obesity in the longitudinal analysis. Note: The figure was based on the logistic regression for abdominal obesity including the interaction term of PM_2.5_ and NDVI adjusting for biomarker measurement year, baseline age, sex, ethnicity, education, marriage, residence, exercise, smoking, alcohol drinking, and GDP per capita. Higher PM_2.5_ was associated with higher probability of AO, and the effect size decreased with the increase of the greenness level for exposure beyond 30 μg/m^3^. Higher NDVI was associated with lower probability of AO and the effect size was stronger under relatively higher PM_2.5_ exposure. We used R package "interactions" to draw the figure.

We found a significant interaction of PM_2.5_ and NDVI on AO (beta estimate of interaction term = − 0.088, *P =* 0.025) and waist circumference (beta estimate of interaction term = − 0.396, *P =* 0.031) (Table 2, Table S4). Higher PM_2.5_ was associated with a higher probability of AO, and the association for exposure beyond 30 μg/m^3^ became stronger with the increase of the greenness level. Higher NDVI was associated with a lower probability of AO and the association was stronger under relatively higher PM_2.5_ exposure (Fig. 2). For the three-way interaction of air pollution, greenness, and gender on metabolic biomarkers, we only found a significant three-way interaction on GSP. In areas with low NDVI, the association strength and direction of PM_2.5_ with GSP in the females were different from males, and applies in areas with high NDVI (Fig. S2).

In the stratified analysis, the association between PM_2.5_ and AO was weaker in areas with high NDVI exposure than areas with low NDVI [OR (95%CI): 1.17 (1.08, 1.28) vs. 1.25 (1.13, 1.39)]. The association between NDVI and AO was only significant in areas with low PM_2.5_ [OR (95%CI): 0.61 (0.52, 0.73)]. PM_2.5_ shown a harmful association with MetS, AO, elevated fasting glucose, and reduced HDL-C only in rural areas [OR (95%CI): 1.18 (1.09, 1.28) for MetS, 1.22 (1.14, 1.30) for AO, 1.08 (1.01, 1.16) for elevated fasting glucose, and 1.15 (1.07, 1.23) for reduced HDL-C], not in urban areas. NDVI’s protective association with AO was a little stronger in urban areas than rural areas. The association between PM_2.5_ with MetS, AO, reduced HDL-C were stronger in the male than female, and the association between NDVI with AO were similar for males and females. The association between PM_2.5_ and MetS as well as its components were all more significant in the population aged younger than 80 compared to those aged 80 or older. NDVI was still not associated with MetS in the two different age groups, but had a stronger association with AO in those younger than 80 (Table 3).

**Table 3:** The association between the greenness and air pollution with the metabolic syndrome and the components (Binary outcome) in the longitudinal analysis stratified by PM_2.5_, NDVI, age, sex, and residence^a^

Outcome (Yes vs. No)	3-year average NDVI (0.1 unit)			3-year average PM_**2.5**_ (10 μg/m^**3**^)		
	Subgroup	OR (95% CI)	***p*** value	Subgroup	OR (95% CI)	***p*** value
Abdominal obesity	PM_2.5 _(10 μg/m^3^) < 5.32	0.61 (0.52, 0.73)	< 0.001	NDVI (0.1 unit) < 5.24	1.25 (1.13, 1.39)	< 0.001
Elevated fasting glucose		0.91 (0.77, 1.06)	0.224		0.98 (0.88, 1.08)	0.625
Hypertension		0.94 (0.81, 1.10)	0.433		1.02 (0.91, 1.14)	0.767
Hypertriglyceridemia		0.89 (0.73, 1.08)	0.238		0.91 (0.79, 1.06)	0.241
Reduced HDL-C		0.98 (0.83, 1.15)	0.784		1.11 (0.99, 1.23)	0.064
MetS		0.82 (0.69, 0.97)	0.021		1.12 (1.00, 1.26)	0.051
Abdominal obesity	PM_2.5 _(10 μg/m^3^) ≥ 5.32	0.99 (0.85, 1.15)	0.893	NDVI (0.1 unit) ≥5.24	1.17 (1.08, 1.28)	< 0.001
Elevated fasting glucose		0.99 (0.86, 1.15)	0.911		1.07 (0.98, 1.18)	0.123
Hypertension		0.96 (0.81, 1.15)	0.679		1.01 (0.92, 1.10)	0.838
Hypertriglyceridemia		1.16 (0.92, 1.45)	0.203		1.05 (0.93, 1.18)	0.423
Reduced HDL-C		1.04 (0.90, 1.20)	0.637		1.06 (0.97, 1.16)	0.187
MetS		1.06 (0.91, 1.24)	0.441		1.13 (1.02, 1.25)	0.015
Abdominal obesity	Urban	0.76 (0.62, 0.93)	0.007	Urban	1.07 (0.88, 1.31)	0.493
Elevated fasting glucose		0.90 (0.73, 1.10)	0.297		0.92 (0.73, 1.15)	0.450
Hypertension		1.09 (0.88, 1.34)	0.438		1.02 (0.80, 1.30)	0.848
Hypertriglyceridemia		1.08 (0.84, 1.37)	0.549		1.05 (0.80, 1.38)	0.720
Reduced HDL-C		1.09 (0.89, 1.34)	0.422		0.96 (0.77, 1.19)	0.706
MetS		1.00 (0.82, 1.22)	0.984		1.01 (0.80, 1.28)	0.923
Abdominal obesity	Rural	0.82 (0.72, 0.93)	0.003	Rural	1.22 (1.14, 1.30)	< 0.001
Elevated fasting glucose		0.94 (0.83, 1.06)	0.292		1.08 (1.01, 1.16)	0.024
Hypertension		0.96 (0.84, 1.09)	0.530		0.99 (0.92, 1.06)	0.742
Hypertriglyceridemia		0.98 (0.82, 1.16)	0.800		1.05 (0.95, 1.15)	0.370
Reduced HDL-C		0.95 (0.84, 1.07)	0.371		1.15 (1.07, 1.23)	< 0.001
MetS		0.91 (0.80, 1.04)	0.150		1.18 (1.09, 1.28)	< 0.001
Abdominal obesity	Male	0.78 (0.67, 0.92)	0.003	Male	1.37 (1.22, 1.53)	< 0.001
Elevated fasting glucose		0.95 (0.81, 1.10)	0.464		1.05 (0.95, 1.15)	0.334
Hypertension		1.07 (0.92, 1.25)	0.373		1.05 (0.96, 1.14)	0.336
Hypertriglyceridemia		1.09 (0.88, 1.36)	0.420		1.03 (0.90, 1.18)	0.667
Reduced HDL-C		0.95 (0.82, 1.11)	0.553		1.17 (1.04, 1.32)	0.008
MetS		0.97 (0.81, 1.16)	0.751		1.22 (1.08, 1.39)	0.002
Abdominal obesity	Female	0.79 (0.68, 0.92)	0.002	Female	1.11 (1.02, 1.20)	0.011
Elevated fasting glucose		0.91 (0.79, 1.05)	0.201		1.06 (0.97, 1.16)	0.183
Hypertension		0.95 (0.81, 1.11)	0.525		0.96 (0.87, 1.06)	0.392
Hypertriglyceridemia		0.96 (0.80, 1.14)	0.614		1.04 (0.92, 1.18)	0.500
Reduced HDL-C		0.98 (0.85, 1.13)	0.791		1.11 (1.02, 1.20)	0.012
MetS		0.91 (0.80, 1.05)	0.199		1.11 (1.02, 1.22)	0.018
Abdominal obesity	Age < 80	0.75 (0.63, 0.89)	0.001	Age < 80	1.26 (1.14, 1.40)	< 0.001
Elevated fasting glucose		0.97 (0.82, 1.14)	0.728		1.10 (0.99, 1.22)	0.067
Hypertension		0.98 (0.84, 1.15)	0.816		1.05 (0.95, 1.16)	0.326
Hypertriglyceridemia		1.04 (0.85, 1.27)	0.699		1.13 (1.00, 1.29)	0.048
Reduced HDL-C		0.86 (0.74, 1.01)	0.065		1.23 (1.10, 1.37)	< 0.001
MetS		0.95 (0.80, 1.12)	0.513		1.27 (1.13, 1.42)	< 0.001
Abdominal obesity	Age ≥ 80	0.82 (0.71, 0.94)	0.005	Age ≥ 80	1.16 (1.07, 1.26)	< 0.001
Elevated fasting glucose		0.90 (0.79, 1.03)	0.128		1.03 (0.94, 1.12)	0.546
Hypertension		1.00 (0.86, 1.17)	0.968		0.97 (0.89, 1.06)	0.473
Hypertriglyceridemia		1.00 (0.82, 1.21)	0.966		0.94 (0.83, 1.07)	0.362
Reduced HDL-C		1.06 (0.92, 1.21)	0.425		1.10 (1.01, 1.19)	0.022
MetS		0.93 (0.81, 1.07)	0.306		1.09 (0.99, 1.20)	0.078

^a^All models adjusted for biomarker measurement year, baseline age, sex, ethnicity, education, marriage, residence, exercise, smoking, alcohol drinking, and GDP per capita

## Discussion

We found air pollution could increase the risk of MetS, AO, and reduced HDL-C while residential greenness could decrease the risk of AO. We further identified an environment-environment interaction of air pollution-greenness on AO. The association strength for air pollution decreased along with the increase of greenness. The association for greenness was stronger under high-level air pollution exposure than that under low-level air pollution.

Two recent meta-analysis studies on air pollution and MetS showed inconsistent findings. One found PM_2.5_ (per 10 μg/m^3^ increase) was not significantly associated with MetS prevalence [OR (95% CI): 1.34 (0.96, 1.89), *P* = 0.09] or MetS incidence [Hazard ratio (HR): 2.78 (95% CI: 0.70, 11.02), *P* = 0.15] [8], while another one found annual PM_2.5_ (per 5 μg/m^3^ increase) was associated with 14% of MetS risk increase [Risk Ratio (RR): 1.14 (95% CI: 1.03, 1.25)] [9]. The included studies reported associations of different sizes in varied areas. Some studies were conducted in areas with a mean PM_2.5_ higher than 50 μg/m^3^. A study in northern rural China reported the adjusted OR of MetS for per 5 μg/m^3^ increment in PM_2.5_ was 1.42 (95% CI: 1.36, 1.49) [11], while another study only found borderline associations and reported the adjusted odds ratio of MetS per 10 μg/m3 increment in PM_2.5_ was 1.09 (95% CI: 1.00, 1.18) in northern urban China [10]. A Korean national cohort found ﻿PM_2.5_ level was significantly associated with a higher risk for developing MetS [HR (95% CI): 1.07 (1.03, 1.11)] [27]. Some studies were conducted in areas with a mean PM_2.5_ lower than 50 μg/m^3^. The study in Saudi Arabian population in Jeddah observed ﻿a significant association between a 10 μg/m^3^ increase in PM_2.5_ and increased risks for MetS [RR (95% CI): 1.12 (1.06, 1.19)] [28]. Another study in the highly urbanized German Ruhr Area reported the OR of per interquartile range (IQR = 1.5 μg/m^3^) PM_2.5_ was 1.04 (95% CI: 0.92, 1.17) for MetS prevalence and 1.21 (95% CI: 0.99, 1.48) for MetS incidence [29]. A 1-μg/m^3^ increase of PM_2.5_ was associated with a higher risk of developing MetS [HR (95% CI): 1.27 (1.06, 1.52)] in an US older men cohort [27]. We found PM_2.5_ was only significantly associated with MetS in rural areas [OR (95%CI) for 10 μg/m3 increment in PM_2.5_: 1.18 (1.09, 1.28)], and not in urban populations. More studies on air pollution-MetS risk association, especially in low−/middle-income countries, are warranted.

There are a few meta-analyses demonstrated the association between PM_2.5_ and MetS composition biomarker: long-term exposure of PM_2.5_ was associated with a higher level of BMI with the pooled β (95% CI) of 0.34 (0.30, 0.38) per 10 mg/m3 increment [30], higher type 2 diabetes incidence [HR (95% CI): 1.10 (1.04, 1.17) per 10 μg/m3 increment] [6], and higher hypertension prevalence [OR (95% CI):1.05 (1.01, 1.09)] [31]. A few studies found air pollutants only significantly associated with TC, not with HDL-C or TG [5]. A previous CLHLS study reported higher 3-year average exposure to PM_2.5_ was associated with higher fasting blood glucose [32]. In our research, we also found higher PM_2.5_ associated with AO, reduced HDL-C and elevated fasting glucose, which was robust among different age and sex groups. However, we only saw PM_2.5_ increased the risk for elevated fasting glucose in rural areas, and risk for hypertriglyceridemia in the population aged younger than 80. We found no significant association between PM_2.5_ and hypertension.

The negative association between greenness and MetS tended to be insignificant in the elderly based on previous studies, which congruent to our observation. KORA F4/FF4 cohort in German found a negative association between greenness and MetS in both cross-sectional and longitudinal analysis in German but both were insignificant [14]. The 33CCHS conducted in northern urban China found the adjusted OR of MetS per IQR increase in 500 m buffer NDVI of August was 0.81 (95% CI: 0.70, 0.93) for the total population aged 18–74 years, but the association disappeared in subgroup participants aged ≥65 [13]. Whitehall II study in the UK (aged 45–69 years at baseline) found a significant negative association [12]. We did not find a significant association of NDVI on MetS in any subgroup in urban or rural areas, for female or male, aged from 65 to 80 or older than 80.

For MetS composition biomarker, a recent meta-analysis showed higher NDVI was associated with lower odds of overweight/obesity [OR (95% CI): 0.88 (0.84, 0.91)], and most studies were from developed nations (88%) [33]. We also found NDVI associated with lower odds of AO. The possible pathway can be that green spaces could decrease sympathetic nervous system activation [34]. A study in urban northeastern China found higher greenness was consistently associated with lower TC, TG, LDL-C levels, higher HDL-C levels [35], and lower fasting glucose levels [36]. We also found greenness negatively associated with TC, LDL-C, but not associated with TG, HDL-C, or fasting glucose.

We found PM_2.5_ and NDVI were both associated with the metabolic biomarkers. The association varied in different age, sex, and residence categories. PM_2.5_ inhalation could cause pulmonary and systemic inflammation. According to the animal findings, rats that were exposed to Beijing’s highly polluted air experienced the following changes: perivascular and peribronchial inflammation in the lungs, increased tissue and systemic oxidative stress, dyslipidemia, and enhanced proinflammatory status of epididymal fat. TLR2/4-dependent inflammatory activation and lipid oxidation in the lung can spill over systemically, leading to metabolic dysfunction and weight gain [37]. The pathways linking greenness to health include physical activity (50 studies), air pollution (43 studies), social interaction/cohesion (27 studies), mental health/stress/well-being (17 studies), perceived greenness/use (16 studies), and physical health/biomarker (14 studies) and other factors according to the latest review of previous empirical studies [38]. Greenness may decrease the risk for obesity by promoting exercise. Greenness and air pollution may act in separate pathways since our two exposure models showed no major mediation effect according to the similar estimates of the single exposure and two-exposure models.

For the relationship between air pollution and greenness, a longitudinal study in China found a significant interaction between PM_2.5_ and NDVI on all-cause mortality, and individuals living in areas with more greenness appear to be affected more by air pollution, but it showed no monotonic trend [16]. An ecological study in Greece found a significant inverse interaction between PM_2.5_ and NDVI on cardiovascular mortality with the PM_2.5_ effects decreasing in areas with higher greenery, and they found no interaction on natural-cause mortality [39]. Previous studies have related both greenness and PM_2.5_ with metabolic syndrome and biomarkers. However, most studies only considered PM_2.5_ as a mediator of greenness. There has been no study reported on the interaction of air pollution and greenness on metabolic biomarkers. We reported NDVI had a significant interaction with PM_2.5_ on AO, but no interaction on metabolic syndrome.

Our study has several strengths. First, our cohort has a relatively older mean age than previous studies, and it has a large sample of centenarians which is rare in the world. Secondly, a limited number of studies focused on greenness and the multiple exposures of both air pollution and greenness. While individual studies on environmental predictors exist, ours is a novel approach to assessing the interaction of air pollution and greenness on metabolic syndrome biomarkers. Third, many previous studies were conducted in specific regions like rural or urban areas. We identified high-risk vulnerable older adults from different geographic regions of China. Fourth, we had repeat measurements of a variety of individual metabolic biomarkers. Fifth, we calculated the greenness and air pollution level at the individual residence level, and we tested different exposure time windows before the health outcome. We also surveyed a wide range of lifestyle and district factors to adjust for possible confounding.

There are several limitations to our study. The specific oldest-old population also limited the generalizability of our findings. Those who were lost to follow-up were older, with a possible selection bias. Thus, we did sensitivity analysis only for those with at least one follow-up, and the results persisted. We lacked the exposure data from 2015 to 2017 and used the same exposure as the 2014 wave for the 2017 wave. We found this should not affect our results much since the trend of PM_2.5_ across 2008–2014 was steady within each area. The sensitivity analysis showed no significant difference among one-year, three-year, and five-year exposure windows. There is also no extensive heterogeneity of PM_2.5_ measurement among participants within each area. This possible misclassification usually attenuates the association to null, which means the exposure of higher resolution may show a stronger association with the health outcomes. In addition, we have no indoor air pollution measurements or greenness accessibility data to account for the dynamic personal exposure, which limited the accuracy of the exposure measurement. For the outcome, we lack the metabolism medication information to better define the metabolic syndrome, which may cause underestimating MetS prevalence. We presented the real-world observational evidence, and there may be residual confounding like the diet. We conducted multiple comparisons without correction, for which we exercised caution by presenting confidence intervals and exact *p*-value.

## Conclusions

Our findings contributed to the evidence of harmful association of PM_2.5_ and protective association of NDVI with specific MetS components in an oldest-old population, newly identified a significant interaction between PM_2.5_ and NDVI on AO, and demonstrated the difference between urban and rural areas. Other than the personal actionable lifestyle risk factors, it is also necessary to incorporate environmental determinants into metabolic diseases prevention. This study emphasized the importance of green space planning in urban areas and air pollution mitigation in rural areas to decrease the CVD burden contributed by MetS biomarkers for the policymakers. Further studies can examine if PM_2.5_ and NDVI only interact or if their effect can counteract each other and explore the underlying biology pathway.

## Supplementary Information

**Additional file 1: Table S1.** Population characteristics between those followed up and lost follow-up.

**Additional file 2: Table S2.** Baseline population characteristics across different counties.

**Additional file 3: Table S3. **The association between the greenness and air pollution with the 2012 baseline metabolic biomarkers (binary outcome).

**Additional file 4: Table S4. **The association between the greenness and air pollution with the metabolic biomarkers (continuous outcome) in the longitudinal analysis.

**Additional file 5: Table S5.** The association between air pollution with the metabolic biomarkers (One-year and five-year exposure) in the longitudinal analysis.

**Additional file 6: Table S6.** The association between greenness, air pollution with the metabolic biomarkers among the participants with at least one follow-up.

**Additional file 7: Table S7.** The association between the greenness and air pollution with the metabolic syndrome and the components (binary outcome) in the longitudinal analysis using the Joint Interim Societies’ definition of MetS for Chinese populations.

**Additional file 8: Figure S1.** Study population.

**Additional file 9: Figure S2.** The three-way interaction model of PM_2.5_, NDVI, and gender on glycated serum protein (GSP).

## Data Availability

The CLHLS datasets are available upon request to the public from the Peking University Open Research Data on CLHLS (http://opendata.pku.edu.cn/dataverse/CHADS).

## Abbreviations

PM_2.5_: Fine particulate matter
MetS: Metabolic syndrome
CLHLS: Chinese Longitudinal Healthy Longevity Survey
NDVI: Normalized Difference Vegetation Index
GSP: Glycated serum protein
TC: Total cholesterol
TG: Triglyceride
HDL-C: High-density lipoprotein cholesterol
LDL-C: Low-density lipoprotein cholesterol
AO: Abdominal obesity
SBP: Systolic blood pressure
DBP: Diastolic blood pressure
GEE: Generalized estimating equations
OR: Odds ratio
CI: Confidence interval
IQR: Interquartile range
