# Supplementary material for: Air pollution, residential greenness, and metabolic dysfunction biomarkers: analyses in the Chinese Longitudinal Healthy Longevity Survey
Source: BMC Public Health. 2022 May 4;22:885. doi: 10.1186/s12889-022-13126-8 (PMC9066955; doi:10.1186/s12889-022-13126-8)
Supplement: Supplementary file 1 — Additional file 1: Table S1. Population characteristics between those followed up and lost follow-up. [file 12889_2022_13126_MOESM1_ESM.docx]

**Table S1. Population characteristics of those followed up and lost follow-up**

| **Variables** | **Follow-up** | | | **Overall** |
| --- | --- | --- | --- | --- |
|  | **followed up (N=3389)** | **Lost follow up (N=1876)** | **P value** | **(N=5265)** |
| **3-year average NDVI: mean (SD) (0.1 unit)** | 4.97 (0.962) | 5.04 (1.05) | 0.018 | 5.00 (0.993) |
| **3-year average PM_2.5_: mean (SD) (10 μg/m³)** | 4.92 (1.51) | 4.80 (1.55) | 0.005 | 4.88 (1.52) |
| **GDP per capita in 2012: mean (SD) (10,000 RMB)** | 4.14 (3.47) | 4.74 (4.48) | <0.001 | 4.35 (3.87) |
| **Sex: n(%)** |  |  |  |  |
| Male | 1646 (48.6%) | 784 (41.8%) | <0.001 | 2430 (46.2%) |
| Female | 1743 (51.4%) | 1092 (58.2%) |  | 2835 (53.8%) |
| **Age: mean (SD)** | 83.4 (12.0) | 89.6 (11.6) | <0.001 | 85.6 (12.2) |
| **Schooling year: n(%)** |  |  |  |  |
| No formal education | 1974 (58.2%) | 1284 (68.4%) | <0.001 | 3258 (61.9%) |
| 1-6 years education | 1065 (31.4%) | 450 (24.0%) |  | 1515 (28.8%) |
| >6 years education | 350 (10.3%) | 142 (7.6%) |  | 492 (9.3%) |
| **Ethnicity: n(%)** |  |  |  |  |
| Han | 3123 (92.2%) | 1737 (92.6%) | 0.604 | 4860 (92.3%) |
| Other | 266 (7.8%) | 139 (7.4%) |  | 405 (7.7%) |
| **Residence: n(%)** |  |  |  |  |
| Urban | 560 (16.5%) | 328 (17.5%) | 0.394 | 888 (16.9%) |
| Rural | 2829 (83.5%) | 1548 (82.5%) |  | 4377 (83.1%) |
| **Marriage: n(%)** |  |  |  |  |
| Currently married | 1479 (43.6%) | 555 (29.6%) | <0.001 | 2034 (38.6%) |
| not married | 1910 (56.4%) | 1321 (70.4%) |  | 3231 (61.4%) |
| **Exercise: n(%)** |  |  |  |  |
| Never | 2766 (81.6%) | 1545 (82.4%) | 0.012 | 4311 (81.9%) |
| Former | 66 (1.9%) | 57 (3.0%) |  | 123 (2.3%) |
| Current | 557 (16.4%) | 274 (14.6%) |  | 831 (15.8%) |
| **Smoking: n(%)** |  |  |  |  |
| Never | 2510 (74.1%) | 1459 (77.8%) | <0.001 | 3969 (75.4%) |
| Former | 281 (8.3%) | 154 (8.2%) |  | 435 (8.3%) |
| <20 times/day | 318 (9.4%) | 168 (9.0%) |  | 486 (9.2%) |
| ≥20 times/day | 280 (8.3%) | 95 (5.1%) |  | 375 (7.1%) |
| **Alcohol: n(%)** |  |  |  |  |
| Never | 2591 (76.5%) | 1513 (80.7%) | <0.001 | 4104 (77.9%) |
| Former | 192 (5.7%) | 108 (5.8%) |  | 300 (5.7%) |
| ≤14g/d(female) 28(male) | 203 (6.0%) | 97 (5.2%) |  | 300 (5.7%) |
| >14g/d(female) 28(male) | 403 (11.9%) | 158 (8.4%) |  | 561 (10.7%) |
