# Supplementary material for: Air pollution, residential greenness, and metabolic dysfunction biomarkers: analyses in the Chinese Longitudinal Healthy Longevity Survey
Source: BMC Public Health. 2022 May 4;22:885. doi: 10.1186/s12889-022-13126-8 (PMC9066955; doi:10.1186/s12889-022-13126-8)
Supplement: Supplementary file 2 — Additional file 2: Table S2. Baseline population characteristics across different counties. [file 12889_2022_13126_MOESM2_ESM.docx]

**Table S2. Baseline population characteristics across different counties**

| **variables** | **Rudong** | **Laizhou** | **Xiayi** | **Zhongxiang** | **Mayang** | **Sanshui** | **Yongfu** | **Chengmai** | **Overall** |
| --- | --- | --- | --- | --- | --- | --- | --- | --- | --- |
|  | **(N=231)** | **(N=276)** | **(N=464)** | **(N=212)** | **(N=104)** | **(N=95)** | **(N=112)** | **(N=261)** | **(N=1755)** |
| **3-year average NDVI: mean (SD) (0.1 unit)** | 5.37 (0.588) | 3.95 (0.751) | 5.23 (0.563) | 4.75 (1.13) | 5.20 (0.640) | 3.36 (0.807) | 5.02 (0.537) | 5.26 (0.698) | 4.88 (0.937) |
| **3-year average PM_2.5_: mean (SD) (10 μg/m³)** | 5.81 (0.0527) | 5.23 (0.0210) | 6.42 (0.0167) | 5.39 (0.0695) | 3.90 (0) | 4.18 (0.0838) | 3.73 (0.0732) | 1.83 (0.0268) | 4.90 (1.53) |
| **GDP per capita in 2012: mean (SD) (10,000 RMB)** | 4.84 (0) | 6.75 (0) | 1.75 (0) | 2.76 (0) | 1.37 (0) | 18.8 (0) | 3.51 (0) | 3.61 (0) | 4.35 (3.87) |
| **Sex: n(%)** |  |  |  |  |  |  |  |  |  |
| Male | 96 (41.6) | 133 (48.2) | 219 (47.2) | 98 (46.2) | 45 (43.3) | 44 (46.3) | 55 (49.1) | 120 (46.0) | 810 (46.2) |
| Female | 135 (58.4) | 143 (51.8) | 245 (52.8) | 114 (53.8) | 59 (56.7) | 51 (53.7) | 57 (50.9) | 141 (54.0) | 945 (53.8) |
| **Age: mean (SD)** | 87.0 (11.5) | 84.9 (11.7) | 85.5 (13.4) | 85.2 (11.4) | 84.8 (12.9) | 87.1 (11.0) | 85.8 (12.4) | 85.2 (11.9) | 85.6 (12.2) |
| **Age group: n(%)** |  |  |  |  |  |  |  |  |  |
| 65 to 79 | 69 (29.9) | 96 (34.8) | 181 (39.0) | 72 (34.0) | 42 (40.4) | 24 (25.3) | 38 (33.9) | 94 (36.0) | 616 (35.1) |
| 80 to 89 | 56 (24.2) | 71 (25.7) | 80 (17.2) | 66 (31.1) | 24 (23.1) | 29 (30.5) | 27 (24.1) | 74 (28.4) | 427 (24.3) |
| 90 to 99 | 51 (22.1) | 62 (22.5) | 79 (17.0) | 38 (17.9) | 20 (19.2) | 25 (26.3) | 21 (18.8) | 43 (16.5) | 339 (19.3) |
| ≥100 | 55 (23.8) | 47 (17.0) | 124 (26.7) | 36 (17.0) | 18 (17.3) | 17 (17.9) | 26 (23.2) | 50 (19.2) | 373 (21.3) |
| **Schooling year: n(%)** |  |  |  |  |  |  |  |  |  |
| No formal education | 147 (63.6) | 122 (44.2) | 346 (74.6) | 141 (66.5) | 59 (56.7) | 49 (51.6) | 56 (50.0) | 166 (63.6) | 1086 (61.9) |
| 1-6 years education | 68 (29.4) | 128 (46.4) | 84 (18.1) | 57 (26.9) | 30 (28.8) | 38 (40.0) | 42 (37.5) | 58 (22.2) | 505 (28.8) |
| >6 years education | 16 (6.9) | 26 (9.4) | 34 (7.3) | 14 (6.6) | 15 (14.4) | 8 (8.4) | 14 (12.5) | 37 (14.2) | 164 (9.3) |
| **Ethnicity: n(%)** |  |  |  |  |  |  |  |  |  |
| Han | 231 (100) | 275 (99.6) | 459 (98.9) | 205 (96.7) | 1 (1.0) | 93 (97.9) | 100 (89.3) | 256 (98.1) | 1620 (92.3) |
| Other | 0 (0) | 1 (0.4) | 5 (1.1) | 7 (3.3) | 103 (99.0) | 2 (2.1) | 12 (10.7) | 5 (1.9) | 135 (7.7) |
| **Marriage: n(%)** |  |  |  |  |  |  |  |  |  |
| Currently married | 82 (35.5) | 131 (47.5) | 169 (36.4) | 84 (39.6) | 42 (40.4) | 34 (35.8) | 32 (28.6) | 104 (39.8) | 678 (38.6) |
| not married | 149 (64.5) | 145 (52.5) | 295 (63.6) | 128 (60.4) | 62 (59.6) | 61 (64.2) | 80 (71.4) | 157 (60.2) | 1077 (61.4) |
| **Exercise: n(%)** |  |  |  |  |  |  |  |  |  |
| Never | 208 (90.0) | 188 (68.1) | 420 (90.5) | 197 (92.9) | 85 (81.7) | 51 (53.7) | 98 (87.5) | 190 (72.8) | 1437 (81.9) |
| Former | 2 (0.9) | 4 (1.4) | 4 (0.9) | 2 (0.9) | 3 (2.9) | 5 (5.3) | 2 (1.8) | 19 (7.3) | 41 (2.3) |
| Current | 21 (9.1) | 84 (30.4) | 40 (8.6) | 13 (6.1) | 16 (15.4) | 39 (41.1) | 12 (10.7) | 52 (19.9) | 277 (15.8) |
| **Smoking: n(%)** |  |  |  |  |  |  |  |  |  |
| Never | 166 (71.9) | 204 (73.9) | 375 (80.8) | 180 (84.9) | 71 (68.3) | 61 (64.2) | 78 (69.6) | 188 (72.0) | 1323 (75.4) |
| Former | 32 (13.9) | 23 (8.3) | 28 (6.0) | 8 (3.8) | 9 (8.7) | 7 (7.4) | 9 (8.0) | 29 (11.1) | 145 (8.3) |
| <20 times/day | 26 (11.3) | 26 (9.4) | 36 (7.8) | 14 (6.6) | 15 (14.4) | 19 (20.0) | 12 (10.7) | 14 (5.4) | 162 (9.2) |
| ≥20 times/day | 7 (3.0) | 23 (8.3) | 25 (5.4) | 10 (4.7) | 9 (8.7) | 8 (8.4) | 13 (11.6) | 30 (11.5) | 125 (7.1) |
| **Alcohol: n(%)** |  |  |  |  |  |  |  |  |  |
| Never | 152 (65.8) | 185 (67.0) | 403 (86.9) | 185 (87.3) | 74 (71.2) | 83 (87.4) | 68 (60.7) | 218 (83.5) | 1368 (77.9) |
| Former | 21 (9.1) | 16 (5.8) | 10 (2.2) | 3 (1.4) | 14 (13.5) | 8 (8.4) | 15 (13.4) | 13 (5.0) | 100 (5.7) |
| ≤14g/d(female) 28(male) | 23 (10.0) | 25 (9.1) | 17 (3.7) | 10 (4.7) | 8 (7.7) | 3 (3.2) | 4 (3.6) | 10 (3.8) | 100 (5.7) |
| >14g/d(female) 28(male) | 35 (15.2) | 50 (18.1) | 34 (7.3) | 14 (6.6) | 8 (7.7) | 1 (1.1) | 25 (22.3) | 20 (7.7) | 187 (10.7) |
| **TC: mean (SD) (mmol/L)** | 4.10 (1.04) | 4.68 (1.04) | 4.17 (0.867) | 4.39 (0.902) | 3.66 (0.872) | 4.20 (1.07) | 4.07 (0.807) | 4.54 (0.937) | 4.29 (0.976) |
| **LDL-C: mean (SD) (mmol/L)** | 2.38 (0.825) | 2.92 (0.867) | 2.55 (0.718) | 2.32 (0.727) | 1.94 (0.690) | 2.59 (0.919) | 2.24 (0.653) | 2.80 (0.819) | 2.54 (0.822) |
| **TG: mean (SD) (mmol/L)** | 0.848 (0.576) | 0.910 (0.568) | 0.847 (0.431) | 1.21 (0.841) | 1.20 (0.990) | 1.27 (0.816) | 1.19 (0.708) | 0.968 (0.596) | 0.985 (0.656) |
| **HDL-C: mean (SD) (mmol/L)** | 1.33 (0.372) | 1.35 (0.376) | 1.23 (0.305) | 1.50 (0.386) | 1.18 (0.320) | 1.04 (0.329) | 1.29 (0.379) | 1.30 (0.317) | 1.30 (0.361) |
| **Waist circumference: mean (SD) (centimeter)** | 81.0 (10.3) | 85.9 (11.8) | 80.3 (10.5) | 75.1 (10.9) | 80.4 (10.5) | 77.6 (8.92) | 78.1 (9.09) | 75.4 (8.71) | 79.6 (10.9) |
| **Fasting glucose: mean (SD) (mmol/L)** | 4.62 (1.55) | 4.97 (1.40) | 4.97 (1.84) | 3.32 (1.60) | 5.80 (2.88) | 4.96 (2.24) | 4.12 (1.49) | 4.53 (3.25) | 4.65 (2.16) |
| **SBP: mean (SD) (mmHg)** | 144 (23.6) | 146 (26.2) | 137 (20.0) | 142 (21.5) | 129 (20.7) | 139 (22.1) | 147 (17.3) | 139 (23.9) | 141 (22.8) |
| **DBP: mean (SD) (mmHg)** | 78.3 (11.9) | 81.1 (11.3) | 85.1 (10.8) | 85.4 (11.6) | 73.4 (13.0) | 79.2 (12.0) | 83.5 (10.3) | 75.8 (10.7) | 81.1 (11.9) |
| **Abdominal obesity: n(%)** |  |  |  |  |  |  |  |  |  |
| No | 143 (61.9) | 122 (44.2) | 308 (66.4) | 172 (81.1) | 68 (65.4) | 70 (73.7) | 83 (74.1) | 206 (78.9) | 1172 (66.8) |
| Yes | 88 (38.1) | 154 (55.8) | 156 (33.6) | 40 (18.9) | 36 (34.6) | 25 (26.3) | 29 (25.9) | 55 (21.1) | 583 (33.2) |
| **Elevated fasting glucose: n(%)** |  |  |  |  |  |  |  |  |  |
| No | 198 (85.7) | 222 (80.4) | 375 (80.8) | 201 (94.8) | 62 (59.6) | 68 (71.6) | 98 (87.5) | 224 (85.8) | 1448 (82.5) |
| Yes | 33 (14.3) | 54 (19.6) | 89 (19.2) | 11 (5.2) | 42 (40.4) | 27 (28.4) | 14 (12.5) | 37 (14.2) | 307 (17.5) |
| **Hypertension: n(%)** |  |  |  |  |  |  |  |  |  |
| No | 60 (26.0) | 59 (21.4) | 131 (28.2) | 46 (21.7) | 48 (46.2) | 28 (29.5) | 19 (17.0) | 79 (30.3) | 470 (26.8) |
| Yes | 171 (74.0) | 217 (78.6) | 333 (71.8) | 166 (78.3) | 56 (53.8) | 67 (70.5) | 93 (83.0) | 182 (69.7) | 1285 (73.2) |
| **Hypertriglyceridemia: n(%)** |  |  |  |  |  |  |  |  |  |
| No | 217 (93.9) | 254 (92.0) | 440 (94.8) | 182 (85.8) | 90 (86.5) | 77 (81.1) | 98 (87.5) | 240 (92.0) | 1598 (91.1) |
| Yes | 14 (6.1) | 22 (8.0) | 24 (5.2) | 30 (14.2) | 14 (13.5) | 18 (18.9) | 14 (12.5) | 21 (8.0) | 157 (8.9) |
| **Low HDL-C: n(%)** |  |  |  |  |  |  |  |  |  |
| No | 148 (64.1) | 188 (68.1) | 252 (54.3) | 170 (80.2) | 52 (50.0) | 31 (32.6) | 69 (61.6) | 166 (63.6) | 1076 (61.3) |
| Yes | 83 (35.9) | 88 (31.9) | 212 (45.7) | 42 (19.8) | 52 (50.0) | 64 (67.4) | 43 (38.4) | 95 (36.4) | 679 (38.7) |
| **Mets: n (%)** |  |  |  |  |  |  |  |  |  |
| No | 183 (79.2) | 203 (73.6) | 359 (77.4) | 187 (88.2) | 75 (72.1) | 65 (68.4) | 89 (79.5) | 224 (85.8) | 1385 (78.9) |
| Yes | 48 (20.8) | 73 (26.4) | 105 (22.6) | 25 (11.8) | 29 (27.9) | 30 (31.6) | 23 (20.5) | 37 (14.2) | 370 (21.1) |
| **Residence: n (%)** |  |  |  |  |  |  |  |  |  |
| Urban | 9 (3.9) | 34 (12.3) | 41 (8.8) | 113 (53.3) | 17 (16.3) | 29 (30.5) | 35 (31.2) | 18 (6.9) | 296 (16.9) |
| Rural | 222 (96.1) | 242 (87.7) | 423 (91.2) | 99 (46.7) | 87 (83.7) | 66 (69.5) | 77 (68.8) | 243 (93.1) | 1459 (83.1) |
