# Supplementary material for: Air pollution, residential greenness, and metabolic dysfunction biomarkers: analyses in the Chinese Longitudinal Healthy Longevity Survey
Source: BMC Public Health. 2022 May 4;22:885. doi: 10.1186/s12889-022-13126-8 (PMC9066955; doi:10.1186/s12889-022-13126-8)
Supplement: Supplementary file 3 — Additional file 3: Table S3. The association between the greenness and air pollution with the 2012 baseline metabolic biomarkers (binary outcome). [file 12889_2022_13126_MOESM3_ESM.docx]

**Table S3. The association between the greenness and air pollution with 2012 baseline metabolic biomarkers (Binary outcome)^a^**

| Outcome | Exposure | Greenness single exposure model (0.1 unit increase of NDVI) | |  | PM_2.5_ single exposure model (10 μg/m³ increase of PM_2.5_) | |  | Greenness & PM_2.5_ two exposure model | |  | Centered Greenness & PM_2.5_ interaction model | | |
| --- | --- | --- | --- | --- | --- | --- | --- | --- | --- | --- | --- | --- | --- |
|  |  | OR (95% CI) | p value |  | OR (95% CI) | p value |  | OR (95% CI) | p value |  | Beta | std error | p value |
| Abdominal obesity | NDVI | 0.807 (0.707, 0.921) | 0.002 |  |  |  |  | 0.832 (0.727, 0.952) | 0.007 |  | -0.157 | 0.07 | 0.024 |
| Abdominal obesity | PM_2.5_ |  |  |  | 1.254 (1.159, 1.361) | <0.001 |  | 1.244 (1.149, 1.35) | <0.001 |  | 0.26 | 0.045 | <0.001 |
| Abdominal obesity | NDVI*PM_2.5_ |  |  |  |  |  |  |  |  |  | -0.148 | 0.057 | 0.01 |
| Elevated fasting glucose | NDVI | 0.843 (0.724, 0.987) | 0.031 |  |  |  |  | 0.85 (0.729, 0.996) | 0.042 |  | -0.169 | 0.081 | 0.037 |
| Elevated fasting glucose | PM_2.5_ |  |  |  | 1.079 (0.985, 1.187) | 0.108 |  | 1.072 (0.978, 1.18) | 0.145 |  | 0.054 | 0.049 | 0.268 |
| Elevated fasting glucose | NDVI*PM_2.5_ |  |  |  |  |  |  |  |  |  | 0.084 | 0.063 | 0.183 |
| Hypertension | NDVI | 0.978 (0.85, 1.122) | 0.755 |  |  |  |  | 0.98 (0.851, 1.125) | 0.777 |  | -0.022 | 0.07 | 0.756 |
| Hypertension | PM_2.5_ |  |  |  | 1.013 (0.94, 1.09) | 0.738 |  | 1.012 (0.939, 1.089) | 0.758 |  | -0.01 | 0.041 | 0.801 |
| Hypertension | NDVI*PM_2.5_ |  |  |  |  |  |  |  |  |  | 0.08 | 0.053 | 0.13 |
| Hypertriglyceridemia | NDVI | 1.03 (0.842, 1.274) | 0.778 |  |  |  |  | 1.016 (0.829, 1.258) | 0.881 |  | 0.018 | 0.107 | 0.866 |
| Hypertriglyceridemia | PM_2.5_ |  |  |  | 0.944 (0.841, 1.064) | 0.331 |  | 0.945 (0.841, 1.066) | 0.347 |  | -0.066 | 0.064 | 0.3 |
| Hypertriglyceridemia | NDVI*PM_2.5_ |  |  |  |  |  |  |  |  |  | 0.034 | 0.081 | 0.675 |
| Low HDL-C | NDVI | 1.046 (0.921, 1.191) | 0.49 |  |  |  |  | 1.058 (0.93, 1.205) | 0.396 |  | 0.055 | 0.066 | 0.408 |
| Low HDL-C | PM_2.5_ |  |  |  | 1.062 (0.991, 1.14) | 0.091 |  | 1.065 (0.993, 1.144) | 0.079 |  | 0.058 | 0.038 | 0.129 |
| Low HDL-C | NDVI*PM_2.5_ |  |  |  |  |  |  |  |  |  | 0.02 | 0.05 | 0.686 |
| MetS | NDVI | 0.94 (0.812, 1.091) | 0.409 |  |  |  |  | 0.963 (0.831, 1.121) | 0.626 |  | -0.036 | 0.077 | 0.641 |
| MetS | PM_2.5_ |  |  |  | 1.167 (1.068, 1.281) | 0.001 |  | 1.165 (1.066, 1.278) | 0.001 |  | 0.155 | 0.05 | 0.002 |
| MetS | NDVI*PM_2.5_ |  |  |  |  |  |  |  |  |  | -0.009 | 0.063 | 0.889 |

a. All models adjusted for age, sex, ethnicity, education, marriage, residence, exercise, smoking, alcohol drinking, and GDP per capital in 2012
