# Supplementary material for: Air pollution, residential greenness, and metabolic dysfunction biomarkers: analyses in the Chinese Longitudinal Healthy Longevity Survey
Source: BMC Public Health. 2022 May 4;22:885. doi: 10.1186/s12889-022-13126-8 (PMC9066955; doi:10.1186/s12889-022-13126-8)
Supplement: Supplementary file 4 — Additional file 4: Table S4. The association between the greenness and air pollution with the metabolic biomarkers (continuous outcome) in the longitudinal analysis. [file 12889_2022_13126_MOESM4_ESM.docx]

**Table S4. The association between the greenness and air pollution with the metabolic biomarkers (continuous outcome) in the longitudinal analysis^a,b^**

| Outcome | Exposure | Greenness single exposure model (0.1 unit increase of NDVI) | |  | PM_2.5_ single exposure model (10 μg/m³ increase of PM_2.5_) | |  | Greenness & PM_2.5_ two exposure model | |  | Centered Greenness & PM_2.5_ interaction model | | |
| --- | --- | --- | --- | --- | --- | --- | --- | --- | --- | --- | --- | --- | --- |
|  |  | Mean difference (95% CI) | p value |  | Mean difference (95% CI) | p value |  | Mean difference (95% CI) | p value |  | Beta | std error | p value |
| TC | NDVI | -0.069 (-0.117, -0.021) | 0.005 |  |  |  |  | -0.078 (-0.127, -0.03) | 0.002 |  | -0.079 | 0.025 | 0.002 |
| TC | PM_2.5_ |  |  |  | -0.038 (-0.066, -0.01) | 0.008 |  | -0.043 (-0.071, -0.015) | 0.003 |  | -0.038 | 0.016 | 0.019 |
| TC | NDVI*PM_2.5_ |  |  |  |  |  |  |  |  |  | -0.015 | 0.017 | 0.38 |
| LDL-C | NDVI | -0.076 (-0.12, -0.033) | 0.001 |  |  |  |  | -0.081 (-0.125, -0.037) | <0.001 |  | -0.083 | 0.023 | <0.001 |
| LDL-C | PM_2.5_ |  |  |  | -0.016 (-0.041, 0.009) | 0.207 |  | -0.022 (-0.047, 0.004) | 0.094 |  | -0.013 | 0.015 | 0.383 |
| LDL-C | NDVI*PM_2.5_ |  |  |  |  |  |  |  |  |  | -0.023 | 0.015 | 0.115 |
| TG | NDVI | -0.009 (-0.05, 0.031) | 0.644 |  |  |  |  | -0.009 (-0.05, 0.031) | 0.658 |  | -0.006 | 0.021 | 0.773 |
| TG | PM_2.5_ |  |  |  | 0.002 (-0.017, 0.022) | 0.826 |  | 0.002 (-0.018, 0.021) | 0.876 |  | -0.016 | 0.012 | 0.162 |
| TG | NDVI*PM_2.5_ |  |  |  |  |  |  |  |  |  | 0.047 | 0.014 | 0.001 |
| HDL-C | NDVI | 0.011 (-0.009, 0.031) | 0.271 |  |  |  |  | 0.006 (-0.013, 0.026) | 0.529 |  | 0.005 | 0.01 | 0.588 |
| HDL-C | PM_2.5_ |  |  |  | -0.023 (-0.034, -0.012) | <0.001 |  | -0.022 (-0.034, -0.011) | <0.001 |  | -0.018 | 0.006 | 0.006 |
| HDL-C | NDVI*PM_2.5_ |  |  |  |  |  |  |  |  |  | -0.013 | 0.007 | 0.083 |
| Waist circumference | NDVI | -1.211 (-1.758, -0.664) | <0.001 |  |  |  |  | -0.994 (-1.534, -0.454) | <0.001 |  | -1.022 | 0.278 | <0.001 |
| Waist circumference | PM_2.5_ |  |  |  | 1.115 (0.833, 1.398) | <0.001 |  | 1.048 (0.76, 1.335) | <0.001 |  | 1.199 | 0.161 | <0.001 |
| Waist circumference | NDVI*PM_2.5_ |  |  |  |  |  |  |  |  |  | -0.396 | 0.184 | 0.031 |
| Glucose | NDVI | -0.043 (-0.135, 0.049) | 0.361 |  |  |  |  | -0.023 (-0.113, 0.066) | 0.607 |  | -0.024 | 0.044 | 0.584 |
| Glucose | PM_2.5_ |  |  |  | 0.096 (0.037, 0.155) | 0.001 |  | 0.094 (0.037, 0.152) | 0.001 |  | 0.099 | 0.027 | <0.001 |
| Glucose | NDVI*PM_2.5_ |  |  |  |  |  |  |  |  |  | -0.011 | 0.047 | 0.808 |
| GSP | NDVI | 0.174 (-1.998, 2.346) | 0.875 |  |  |  |  | 0.028 (-2.074, 2.13) | 0.979 |  | -0.073 | 1.051 | 0.945 |
| GSP | PM_2.5_ |  |  |  | -0.706 (-1.966, 0.555) | 0.272 |  | -0.704 (-1.927, 0.519) | 0.259 |  | -0.149 | 0.544 | 0.784 |
| GSP | NDVI*PM_2.5_ |  |  |  |  |  |  |  |  |  | -1.454 | 1.086 | 0.18 |
| SBP | NDVI | -0.569 (-1.786, 0.649) | 0.36 |  |  |  |  | -0.616 (-1.849, 0.617) | 0.328 |  | -0.595 | 0.629 | 0.345 |
| SBP | PM_2.5_ |  |  |  | -0.187 (-0.844, 0.47) | 0.577 |  | -0.229 (-0.894, 0.436) | 0.5 |  | -0.347 | 0.405 | 0.392 |
| SBP | NDVI*PM_2.5_ |  |  |  |  |  |  |  |  |  | 0.309 | 0.484 | 0.523 |
| DBP | NDVI | -0.566 (-1.128, -0.004) | 0.048 |  |  |  |  | -0.344 (-0.913, 0.224) | 0.235 |  | -0.346 | 0.29 | 0.233 |
| DBP | PM_2.5_ |  |  |  | 1.093 (0.772, 1.413) | <0.001 |  | 1.069 (0.746, 1.392) | <0.001 |  | 1.078 | 0.189 | <0.001 |
| DBP | NDVI*PM_2.5_ |  |  |  |  |  |  |  |  |  | -0.023 | 0.216 | 0.915 |

a. Outcome - mmol/L for cholesterol and fasting glucose, μmol/L for GSP, kg/m2 for BMI, centimeter for waist circumference, mmHg for SBP and DBP; Exposure - 0.1 unit of NDVI, 10 μg/m³ of PM_2.5_;

b. All models adjusted for biomarker measurement year, baseline age, sex, ethnicity, education, marriage, residence, exercise, smoking, alcohol drinking, and GDP per capital in 2012.
