# Supplementary material for: Air pollution, residential greenness, and metabolic dysfunction biomarkers: analyses in the Chinese Longitudinal Healthy Longevity Survey
Source: BMC Public Health. 2022 May 4;22:885. doi: 10.1186/s12889-022-13126-8 (PMC9066955; doi:10.1186/s12889-022-13126-8)
Supplement: Supplementary file 5 — Additional file 5: Table S5. The association between air pollution with the metabolic biomarkers (One-year and five-year exposure) in the longitudinal analysis. [file 12889_2022_13126_MOESM5_ESM.docx]

**Table S5. The association between greenness, air pollution with the metabolic biomarkers (One-year and five-year exposure) in the longitudinal analysis**

| **Exposure** | **Outcome (Yes vs. No)** | **1-year average** | | **5-year average** | |
| --- | --- | --- | --- | --- | --- |
|  |  | **OR (95% CI)** | **p value** | **OR (95% CI)** | **p value** |
| **NDVI (0.1 unit of NDVI)** | **Abdominal obesity** | 0.794 (0.714, 0.884) | <0.001 | 0.778 (0.695, 0.871) | <0.001 |
|  | **Elevated fasting glucose** | 0.937 (0.848, 1.036) | 0.206 | 0.932 (0.838, 1.037) | 0.197 |
|  | **Hypertension** | 0.987 (0.888, 1.097) | 0.812 | 0.983 (0.881, 1.097) | 0.765 |
|  | **Hypertriglyceridemia** | 1.029 (0.901, 1.175) | 0.678 | 1.023 (0.89, 1.177) | 0.749 |
|  | **Low HDL-C** | 0.973 (0.879, 1.077) | 0.6 | 0.987 (0.887, 1.097) | 0.804 |
|  | **MetS** | 0.937 (0.843, 1.042) | 0.231 | 0.93 (0.832, 1.039) | 0.197 |
|  |  |  |  |  |  |
| **PM_2.5_ (10 μg/m³)** | **Abdominal obesity** | 1.193 (1.116, 1.274) | <0.001 | 1.197 (1.124, 1.275) | <0.001 |
|  | **Elevated fasting glucose** | 1.088 (1.016, 1.165) | 0.016 | 1.061 (0.996, 1.131) | 0.068 |
|  | **Hypertension** | 0.983 (0.916, 1.055) | 0.63 | 0.992 (0.929, 1.058) | 0.803 |
|  | **Hypertriglyceridemia** | 1.065 (0.968, 1.171) | 0.197 | 1.034 (0.946, 1.13) | 0.461 |
|  | **Low HDL-C** | 1.166 (1.089, 1.249) | <0.001 | 1.138 (1.068, 1.214) | <0.001 |
|  | **MetS** | 1.17 (1.086, 1.261) | <0.001 | 1.156 (1.076, 1.241) | <0.001 |

Note. All models adjusted for biomarker measurement year, baseline age, sex, ethnicity, education, marriage, residence, exercise, smoking, alcohol drinking, and GDP per capital in 2012
