# Supplementary material for: Air pollution, residential greenness, and metabolic dysfunction biomarkers: analyses in the Chinese Longitudinal Healthy Longevity Survey
Source: BMC Public Health. 2022 May 4;22:885. doi: 10.1186/s12889-022-13126-8 (PMC9066955; doi:10.1186/s12889-022-13126-8)
Supplement: Supplementary file 6 — Additional file 6: Table S6. The association between greenness, air pollution with the metabolic biomarkers among the participants with at least one follow-up. [file 12889_2022_13126_MOESM6_ESM.docx]

**Table S6. The association between greenness, air pollution with the metabolic biomarkers among the participants with at least one follow-up**

| **Outcome** | **Exposure** | Greenness single exposure model (0.1 unit increase of NDVI) | |  | PM_2.5_ single exposure model (10 μg/m³ increase of PM_2.5_) | |  | Greenness & PM_2.5_ two exposure model | |  | Centered Greenness & PM_2.5_ interaction model | | |
| --- | --- | --- | --- | --- | --- | --- | --- | --- | --- | --- | --- | --- | --- |
|  |  | OR (95% CI) | p value |  | OR (95% CI) | p value |  | OR (95% CI) | p value |  | Beta | std error | p value |
| Abdominal obesity | NDVI | 0.803 (0.714, 0.904) | <0.001 |  |  |  |  | 0.826 (0.734, 0.93) | 0.002 |  | -0.194 | 0.061 | 0.002 |
| Abdominal obesity | PM_2.5_ |  |  |  | 1.2 (1.116, 1.289) | <0.001 |  | 1.185 (1.102, 1.276) | <0.001 |  | 0.199 | 0.042 | <0.001 |
| Abdominal obesity | NDVI*PM_2.5_ |  |  |  |  |  |  |  |  |  | -0.072 | 0.043 | 0.096 |
| Elevated fasting glucose | NDVI | 0.948 (0.845, 1.063) | 0.36 |  |  |  |  | 0.958 (0.853, 1.076) | 0.469 |  | -0.039 | 0.06 | 0.515 |
| Elevated fasting glucose | PM_2.5_ |  |  |  | 1.059 (0.984, 1.139) | 0.127 |  | 1.055 (0.98, 1.136) | 0.153 |  | 0.035 | 0.042 | 0.402 |
| Elevated fasting glucose | NDVI*PM_2.5_ |  |  |  |  |  |  |  |  |  | 0.044 | 0.045 | 0.327 |
| Hypertension | NDVI | 1.006 (0.892, 1.134) | 0.921 |  |  |  |  | 1.007 (0.893, 1.136) | 0.91 |  | 0.006 | 0.062 | 0.917 |
| Hypertension | PM_2.5_ |  |  |  | 1.004 (0.929, 1.085) | 0.923 |  | 1.004 (0.929, 1.086) | 0.914 |  | 0.006 | 0.047 | 0.898 |
| Hypertension | NDVI*PM_2.5_ |  |  |  |  |  |  |  |  |  | -0.004 | 0.057 | 0.939 |
| Hypertriglyceridemia | NDVI | 1.028 (0.89, 1.186) | 0.708 |  |  |  |  | 1.042 (0.899, 1.207) | 0.587 |  | 0.064 | 0.08 | 0.425 |
| Hypertriglyceridemia | PM_2.5_ |  |  |  | 1.057 (0.956, 1.169) | 0.278 |  | 1.061 (0.958, 1.175) | 0.255 |  | -0.015 | 0.055 | 0.786 |
| Hypertriglyceridemia | NDVI*PM_2.5_ |  |  |  |  |  |  |  |  |  | 0.178 | 0.062 | 0.004 |
| Low HDL-C | NDVI | 0.954 (0.849, 1.072) | 0.433 |  |  |  |  | 0.979 (0.869, 1.104) | 0.732 |  | -0.019 | 0.062 | 0.759 |
| Low HDL-C | PM_2.5_ |  |  |  | 1.164 (1.076, 1.259) | <0.001 |  | 1.162 (1.074, 1.258) | <0.001 |  | 0.108 | 0.044 | 0.014 |
| Low HDL-C | NDVI*PM_2.5_ |  |  |  |  |  |  |  |  |  | 0.114 | 0.048 | 0.017 |
| MetS | NDVI | 0.939 (0.835, 1.055) | 0.29 |  |  |  |  | 0.965 (0.856, 1.089) | 0.565 |  | -0.034 | 0.062 | 0.579 |
| MetS | PM_2.5_ |  |  |  | 1.169 (1.077, 1.268) | <0.001 |  | 1.166 (1.073, 1.266) | <0.001 |  | 0.127 | 0.046 | 0.005 |
| MetS | NDVI*PM_2.5_ |  |  |  |  |  |  |  |  |  | 0.065 | 0.047 | 0.162 |

Note. All models adjusted for biomarker measurement year, baseline age, sex, ethnicity, education, marriage, residence, exercise, smoking, alcohol drinking, and GDP per capital in 2012.
