# Supplementary material for: Air pollution, residential greenness, and metabolic dysfunction biomarkers: analyses in the Chinese Longitudinal Healthy Longevity Survey
Source: BMC Public Health. 2022 May 4;22:885. doi: 10.1186/s12889-022-13126-8 (PMC9066955; doi:10.1186/s12889-022-13126-8)
Supplement: Supplementary file 7 — Additional file 7: Table S7. The association between the greenness and air pollution with the metabolic syndrome and the components (binary outcome) in the longitudinal analysis using the Joint Interim Societies’ definition of MetS for Chinese populations. [file 12889_2022_13126_MOESM7_ESM.docx]

**Table S7. The association between the greenness and air pollution with the metabolic syndrome and the components (binary outcome) in the longitudinal analysis using the ﻿Joint Interim Societies’ definition of MetS for Chinese populations**

| Outcome | Exposure | Greenness single exposure model (0.1 unit increase of NDVI) | |  | PM_2.5_ single exposure model (10 μg/m³ increase of PM_2.5_) | |  | Greenness & PM_2.5_ two exposure model | |  | Centered Greenness & PM_2.5_ interaction model | | |
| --- | --- | --- | --- | --- | --- | --- | --- | --- | --- | --- | --- | --- | --- |
|  |  | OR (95% CI) | p value |  | OR (95% CI) | p value |  | OR (95% CI) | p value |  | Beta | std error | p value |
| Abdominal obesity | NDVI | 0.805 (0.723, 0.895) | <0.001 |  |  |  |  | 0.829 (0.746, 0.922) | 0.001 |  | -0.190 | 0.055 | 0.001 |
| Abdominal obesity | PM_2.5_ |  |  |  | 1.187 (1.118, 1.261) | <0.001 |  | 1.173 (1.103, 1.247) | <0.001 |  | 0.187 | 0.035 | <0.001 |
| Abdominal obesity | NDVI*PM_2.5_ |  |  |  |  |  |  |  |  |  | -0.072 | 0.038 | 0.056 |
| Elevated fasting glucose | NDVI | 0.933 (0.842, 1.035) | 0.192 |  |  |  |  | 0.943 (0.849, 1.048) | 0.277 |  | -0.054 | 0.055 | 0.332 |
| Elevated fasting glucose | PM_2.5_ |  |  |  | 1.061 (0.995, 1.132) | 0.071 |  | 1.057 (0.99, 1.128) | 0.096 |  | 0.027 | 0.037 | 0.464 |
| Elevated fasting glucose | NDVI*PM_2.5_ |  |  |  |  |  |  |  |  |  | 0.076 | 0.042 | 0.073 |
| Hypertension | NDVI | 0.993 (0.892, 1.106) | 0.902 |  |  |  |  | 0.991 (0.889, 1.104) | 0.872 |  | -0.008 | 0.055 | 0.885 |
| Hypertension | PM_2.5_ |  |  |  | 0.99 (0.927, 1.057) | 0.762 |  | 0.989 (0.926, 1.057) | 0.75 |  | -0.015 | 0.039 | 0.696 |
| Hypertension | NDVI*PM_2.5_ |  |  |  |  |  |  |  |  |  | 0.012 | 0.049 | 0.808 |
| Hypertriglyceridemia | NDVI | 1.014 (0.886, 1.16) | 0.843 |  |  |  |  | 1.022 (0.891, 1.173) | 0.752 |  | 0.042 | 0.074 | 0.574 |
| Hypertriglyceridemia | PM_2.5_ |  |  |  | 1.035 (0.946, 1.133) | 0.449 |  | 1.037 (0.947, 1.136) | 0.43 |  | -0.026 | 0.049 | 0.592 |
| Hypertriglyceridemia | NDVI*PM_2.5_ |  |  |  |  |  |  |  |  |  | 0.158 | 0.056 | 0.005 |
| Reduced HDL-C | NDVI | 0.976 (0.88, 1.082) | 0.646 |  |  |  |  | 1 (0.9, 1.111) | 0.998 |  | 0.001 | 0.055 | 0.981 |
| Reduced HDL-C | PM_2.5_ |  |  |  | 1.138 (1.067, 1.213) | <0.001 |  | 1.138 (1.066, 1.214) | <0.001 |  | 0.095 | 0.036 | 0.009 |
| Reduced HDL-C | NDVI*PM_2.5_ |  |  |  |  |  |  |  |  |  | 0.095 | 0.041 | 0.019 |
| MetS | NDVI | 0.938 (0.846, 1.040) | 0.222 |  |  |  |  | 0.963 (0.866, 1.07) | 0.479 |  | -0.038 | 0.054 | 0.484 |
| MetS | PM_2.5_ |  |  |  | 1.147 (1.071, 1.229) | <0.001 |  | 1.144 (1.067, 1.227) | <0.001 |  | 0.114 | 0.039 | 0.003 |
| MetS | NDVI*PM_2.5_ |  |  |  |  |  |  |  |  |  | 0.052 | 0.041 | 0.201 |

Note: a. Joint Interim Societies’ definition of MetS for Chinese populations: (1) Waist circumference ≥85 cm for men or ≥80 cm for women; (2) FBG level ≥100 mg/dL (or 5.6 mmol/L); (3) TG ≥150 mg/dL (or 1.7 mmol/L); (4) HDL-C <40 mg/dL (1.0 mmol/L) for men or <50 mg/dL (1.3 mmol/L) for women; (5) SBP ≥130 mmHg or DBP ≥85 mmHg. b. All models adjusted for biomarker measurement year, baseline age, sex, ethnicity, education, marriage, residence, exercise, smoking, alcohol drinking, and GDP per capital in 2012.
