# Supplementary figures and images for: Air pollution, residential greenness, and metabolic dysfunction biomarkers: analyses in the Chinese Longitudinal Healthy Longevity Survey
Source: BMC Public Health. 2022 May 4;22:885. doi: 10.1186/s12889-022-13126-8 (PMC9066955; doi:10.1186/s12889-022-13126-8)

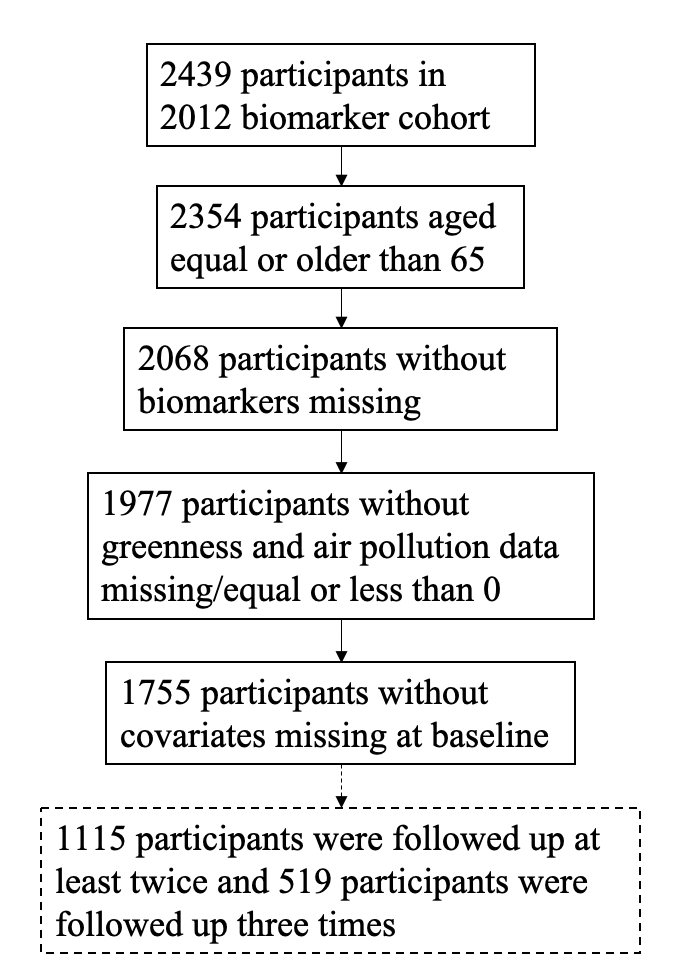

Supplement: Supplementary file 8 — Additional file 8: Figure S1. Study population. [file 12889_2022_13126_MOESM8_ESM.png]

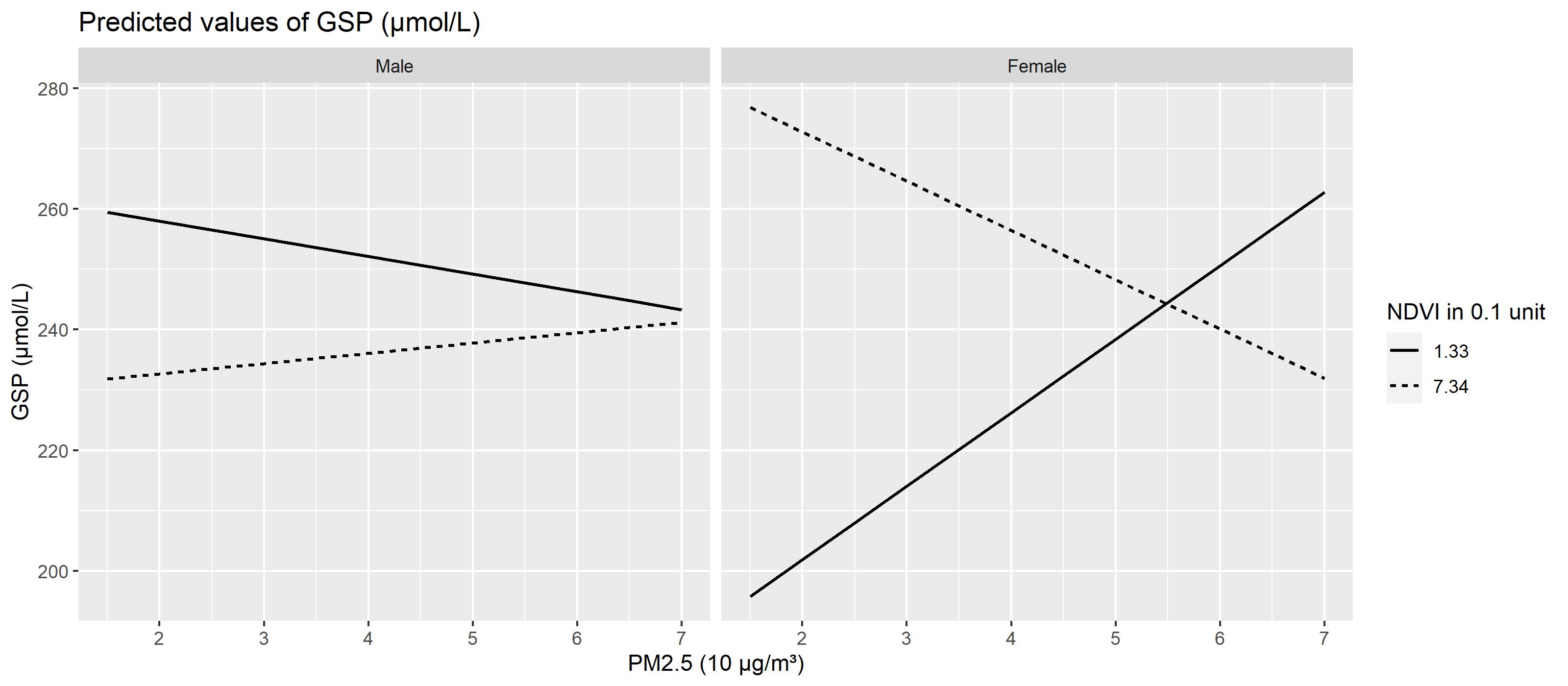

Supplement: Supplementary file 9 — Additional file 9: Figure S2. The three-way interaction model of PM2.5, NDVI, and gender on glycated serum protein (GSP). [file 12889_2022_13126_MOESM9_ESM.jpeg]
